# Age Targeting of Voluntary Medical Male Circumcision Programs Using the Decision Makers’ Program Planning Toolkit (DMPPT) 2.0

**DOI:** 10.1371/journal.pone.0156909

**Published:** 2016-07-13

**Authors:** Katharine Kripke, Marjorie Opuni, Melissa Schnure, Sema Sgaier, Delivette Castor, Jason Reed, Emmanuel Njeuhmeli, John Stover

**Affiliations:** 1 Health Policy Project, Avenir Health, Washington, DC, United States of America; 2 United Nations Joint Programme on HIV/AIDS (UNAIDS), Geneva, Switzerland; 3 Health Policy Project, Futures Group, Washington, DC, United States of America; 4 Bill & Melinda Gates Foundation, Seattle, WA, United States of America; 5 Department of Global Health, University of Washington, Seattle, WA, United States of America; 6 Office of the U.S. Global AIDS Coordinator and Health Diplomacy, Washington, DC, United States of America; 7 USAID, Washington, DC, United States of America; 8 Health Policy Project, Avenir Health, Glastonbury, CT, United States of America; University of Ottawa, CANADA

## Abstract

**Background:**

Despite considerable efforts to scale up voluntary medical male circumcision (VMMC) for HIV prevention in priority countries over the last five years, implementation has faced important challenges. Seeking to enhance the effect of VMMC programs for greatest and most immediate impact, the U. S. President’s Plan for AIDS Relief (PEPFAR) supported the development and application of a model to inform national planning in five countries from 2013–2014.

**Methods and Findings:**

The Decision Makers’ Program Planning Toolkit (DMPPT) 2.0 is a simple compartmental model designed to analyze the effects of client age and geography on program impact and cost. The DMPPT 2.0 model was applied in Malawi, South Africa, Swaziland, Tanzania, and Uganda to assess the impact and cost of scaling up age-targeted VMMC coverage. The lowest number of VMMCs per HIV infection averted would be produced by circumcising males ages 20–34 in Malawi, South Africa, Tanzania, and Uganda and males ages 15–34 in Swaziland. The most immediate impact on HIV incidence would be generated by circumcising males ages 20–34 in Malawi, South Africa, Tanzania, and Uganda and males ages 20–29 in Swaziland. The greatest reductions in HIV incidence over a 15-year period would be achieved by strategies focused on males ages 10–19 in Uganda, 15–24 in Malawi and South Africa, 10–24 in Tanzania, and 15–29 in Swaziland. In all countries, the lowest cost per HIV infection averted would be achieved by circumcising males ages 15–34, although in Uganda this cost is the same as that attained by circumcising 15- to 49-year-olds.

**Conclusions:**

The efficiency, immediacy of impact, magnitude of impact, and cost-effectiveness of VMMC scale-up are not uniform; there is important variation by age group of the males circumcised and countries should plan accordingly.

## Introduction

Notwithstanding important declines in global HIV incidence and AIDS-related mortality [1], HIV/AIDS remains one of the leading causes of death and disability globally [2,3]. At the same time, following more than a decade of significant increases in financing for HIV services in low- and middle-income countries, there has been a leveling off of funding in recent years [1]. In this context, maximizing the use of available resources for highest impact is indispensable [4–7]; not doing so means that money is wasted and, more important, the opportunity is missed to provide services to those in need [8]. There is increasing international commitment to respond more effectively to the epidemic in order to achieve epidemic control by 2030 as exemplified by the United Nations Joint Programme on HIV/AIDS (UNAIDS) Fast-Track initiative [9] and the U.S. President’s Emergency Plan for AIDS Relief (PEPFAR) 2014 plan, “PEPFAR 3.0: Controlling the Epidemic: Delivering on the Promise of an AIDS-free Generation” [10]. There is also growing consensus that to optimize the effect of HIV resources, countries need to scale up evidence-based interventions and focus on the appropriate populations and geographic areas to minimize new HIV infections and AIDS-related deaths [4,6,11].

Voluntary medical male circumcision (VMMC) is a highly effective HIV prevention intervention shown to reduce female-to-male transmission of HIV in three randomized controlled trials [12–14] and recommended in countries with high HIV prevalence and low levels of male circumcision [15]. Modeling was conducted in 2011 to inform the Joint Strategic Action Framework to Accelerate the Scale-Up of Voluntary Medical Male Circumcision for HIV Prevention in Eastern and Southern Africa, outlining VMMC implementation objectives in 14 priority countries [16]. This work suggested that scaling up adult VMMC to reach 80% coverage among males ages 15–49 by 2015 would require completing 20 million circumcisions between 2011 and 2015, with a further 8.4 million circumcisions required between 2016 and 2025 [16,17].

Despite considerable efforts to scale up VMMC over the past five years, as of December 2014, the 14 priority countries had conducted approximately 9 million circumcisions toward the 2015 target of 20 million [18]. Implementation has faced a number of challenges in both the supply of VMMC and the demand for services [19]. Whereas supply has been constrained by policy and planning delays—as well as by financial and human resource limitations—in many places VMMC continues to have low desirability and efforts to generate demand for circumcision have been only partially successful [19,20].

Routine program data accumulated in the 14 priority countries over the past few years show that demand for VMMC services varies by age. Whereas initial rollout of VMMC programs in most countries focused on all males ages 15–49 (who compose the bulk of the sexually active male population), roughly 35% of clients accessing VMMC services were ages 10–14 [18]. This raised the question of how circumcision of 10- to 14-year-olds contributes to the reduction of HIV incidence. In contrast, in most of the 14 countries, men over the age of 25 were accessing services at a lower rate than would be expected based on their distribution in the population, raising the question of the importance of this age group to reducing HIV incidence [21–25].

Given this information and consistent with its goal of focusing the right interventions on the right populations and geographic areas to yield the greatest and most immediate reductions in HIV incidence to achieve epidemic control by 2030 [10], starting in 2013, PEPFAR through the U.S. Agency for International Development (USAID), together with the Bill & Melinda Gates Foundation (BMGF), has supported analyses to inform the prioritization of VMMC scale-up. This work, done in select priority countries in eastern and southern Africa, expands on the first generation of Decision Makers’ Program Planning Toolkit (DMPPT) modeling, which assessed the cost and impact of national and uniform scale-up of circumcision among 15- to 49-year-olds with no variation by age or subnational geographic location [17]. PEPFAR, through USAID, supported the development and application of the DMPPT 2.0 tool, a simple model based in Microsoft Excel to help provide epidemiological impact (HIV infections averted), cost, and cost-effectiveness estimates for VMMC scale-up scenarios focusing on specific client ages and geographic areas. Concurrently, the BMGF financed the elaboration and use of the Age-Structured Model (ASM), a complex simulation model that incorporates VMMC client age and subnational region as well as patterns of sexual mixing and risk behavior. The ASM and its application in Zambia and Zimbabwe are described in this collection [26].

This paper introduces the DMPPT 2.0 tool and assesses the epidemiological impact and cost-effectiveness of VMMC age-targeting strategies in five countries where the model has been applied to date: Malawi, South Africa, Swaziland, Tanzania, and Uganda. Additional details of the country-specific analyses are presented in accompanying country manuscripts [21–25].

## Methods

### DMPPT 2.0 model

DMPPT 2.0 is a simple compartmental model implemented in Microsoft Excel 2010 and designed to analyze the effects of age at circumcision on epidemiological impact and cost. The DMPPT 2.0 model tracks the number of circumcised males beginning at birth and in five-year age groups over time, taking into account age progression and mortality. The model calculates HIV infections averted and discounted VMMC program costs in the population in each year in a user-specified VMMC scale-up strategy (“test scenario”), compared with a baseline scenario in which the male circumcision (MC) prevalence remains constant, based on the most recent population-level survey in the country. The baseline scenario provides the numbers of circumcisions needed to maintain the baseline male circumcision prevalence in each age group as the population of each age group changes over time. The circumcisions in the baseline scenario are assumed to be conducted by traditional circumcisions or by medical male circumcisions that were happening before the initiation of the VMMC program. In the test scenario, the model calculates the number of VMMC program circumcisions needed to increase circumcision prevalence above the baseline levels to reach the user-specified targets. The same number of baseline circumcisions is included in both the baseline and test scenarios, unless the user specifies that a certain percentage of baseline circumcisions are replaced by the VMMC program. Details of the model structure and calculations are provided below.

**Fig 1:**
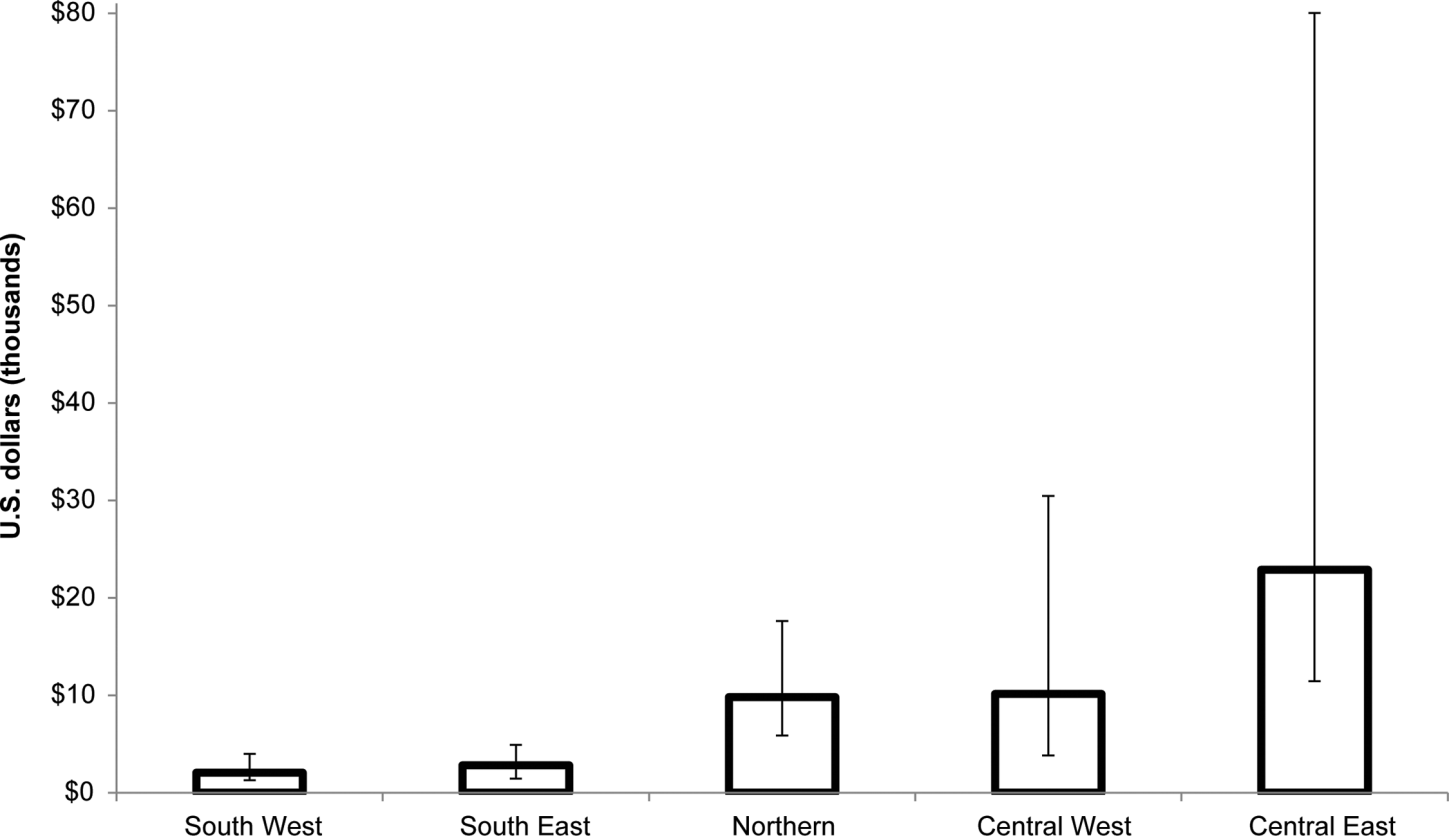
Structure of the DMPPT 2.0 model. μ, non-AIDS mortality; α, AIDS-related mortality; *I*_*u*,*a*,_, HIV incidence of uncircumcised men; *I*_*c*,*a*_, HIV incidence of circumcised men.

The basic structure of the model for both the baseline and test scenarios is shown in Fig 1. The number of circumcised males of age *a* (*M*_*c*,*a*_) is increased by circumcised males aging in from the next younger age group (*M*_*c*,*a-1*_) and by the number of new circumcisions (*NewM*_*c*,*a*_) among the uncircumcised population (*M*_*u*,*a*_) and decreased by mortality (*μ*_*a*_) and by males aging out to the next older age.

Information on the current HIV incidence by age is provided exogenously. It is usually based on national estimates prepared using the Spectrum/AIM model (AIDS Impact Model) under the guidance of UNAIDS. That approach estimates HIV incidence trends for the population of males ages 15–49 by fitting prevalence curves to surveillance and survey data [27] and estimates the age pattern of HIV incidence using incidence rate ratios derived from cohort data [28]. These are the same estimates published in the annual UNAIDS global AIDS reports [1]. Information on the prevalence of male circumcision by age from national surveys prior to initiation of the VMMC program was used to estimate the baseline numbers of circumcisions in each age group in each year, outside of the VMMC program.

HIV incidence among all men of age *a* in year *t* (*I*_*a*,*t*_) is a weighted combination of incidence among circumcised and uncircumcised men.

$$I_{a,t}=I_{c,a,t}\times C_{a,t}+I_{u,a,t}\times (1-C_{a,t})$$ (1)

Where

*I*_*a*,*t*_ = Incidence among all men of age *a* in year *t*

*I*_*c*,*a*,*t*_ = Incidence among circumcised men of age *a* in year *t*

*I*_*u*,*a*,*t*_ = Incidence among uncircumcised men of age *a* in year *t*

*C*_*a*,*t*_ = Circumcision coverage among men of age *a* in year *t*

The ratio of HIV incidence among circumcised men to uncircumcised men is based on three randomized controlled trials [12–14]. We estimate the incidence among circumcised and uncircumcised men by setting incidence among circumcised men equal to incidence among uncircumcised men multiplied by the reduction in incidence due to circumcision.

$$I_{c,a,t}=I_{u,a,t}\;*\;(1-e)$$ (2)

Where

*e* = efficacy of male circumcision

HIV incidence among circumcised men was calculated from the total incidence and the efficacy of circumcision:

$$I_{u,a,t}=\frac{I_{a,t}}{\left(1-C_{a,t=0}\;*\;e\right)}$$ (3)

The number of circumcised men (*M*_*c*,*a*,*t*_) in each age group at time *t* was calculated from the number surviving from the previous year plus new circumcisions and those aging in minus those aging out.

$$M_{c,a,t}=M_{c,a,t-1}\;*\;(1-\mu_{a,t-1})+(NewM_{c,a,t-1}+AgingIn_{a,t-1}-AgingOut_{a,t-1})\;*\;\left(1-\frac{\mu_{a,t-1}}{2}\right)$$ (4)

Where

*μ*_*a*,*t*_ = Mortality among all men of age *a* in year *t*

The number of new male circumcisions at each age (*NewM*_*c*,*a*_) is calculated from the number of males of age *a* (*M*_*a*,*t*_) and coverage target (*C*_*a*,*t*_) minus the number already circumcised.

$$NewM_{c,a,t}=M_{a,t}\;*\;C_{a,t}-(M_{c,a,t}+AgingIn_{a,t}+AgingOut_{a,t})$$ (5)

Both HIV-positive and HIV-negative men are assumed to be circumcised at a rate proportional to their representation in the population. The number of circumcisions to HIV-negative men (*NewM*^*+*^_*c*,*a*_) is the total number of new circumcisions multiplied by the percentage who are not infected, one minus the HIV prevalence (*p*_*a*,*t*_).

$$NewM^{+}{}_{ca,t}=NewM_{c,a,t}\;*\;(1-p_{a,t})$$ (6)

The number of new HIV infections (*NI*) at each age is the sum of new HIV infections to circumcised men and new HIV infections to uncircumcised men.

$$NI_{a,t}=M_{c,a,t}\;*\;(1-p_{a,t})\;*\;I_{c,a,t}+M_{u,a,t}\;*\;(1-p_{a,t})\;*\;I_{u,a,t}$$ (7)

The number of HIV infections averted was calculated by subtracting the number of new HIV infections in the test scenario (*NI*_*t*_) from the number of new HIV infections in the baseline scenario. Since the baseline circumcisions are included in both the baseline and test scenarios, the efficacy of traditional circumcision is canceled out in the model and did not need to be specified. The stream of future HIV infections averted was discounted and summed to produce a total number of HIV infections averted by circumcising a certain number of men, distributed by age and over time. The discounted cumulative number of HIV infections averted was divided by the number of circumcisions performed to estimate the HIV infections averted per VMMC for each age group. HIV infections averted among females due to reduced HIV prevalence among males were estimated using a country-specific ratio to HIV infections averted among circumcised males.

Costs were estimated by multiplying the number of new VMMC program circumcisions performed by two components of cost, each of which may vary by client age group: the cost of the operation and the cost of the recruitment. The cost per HIV infection averted was the sum of the discounted costs of circumcisions in each future year divided by the discounted cumulative future HIV infections averted.

### Model limitations

**Fig 2:**
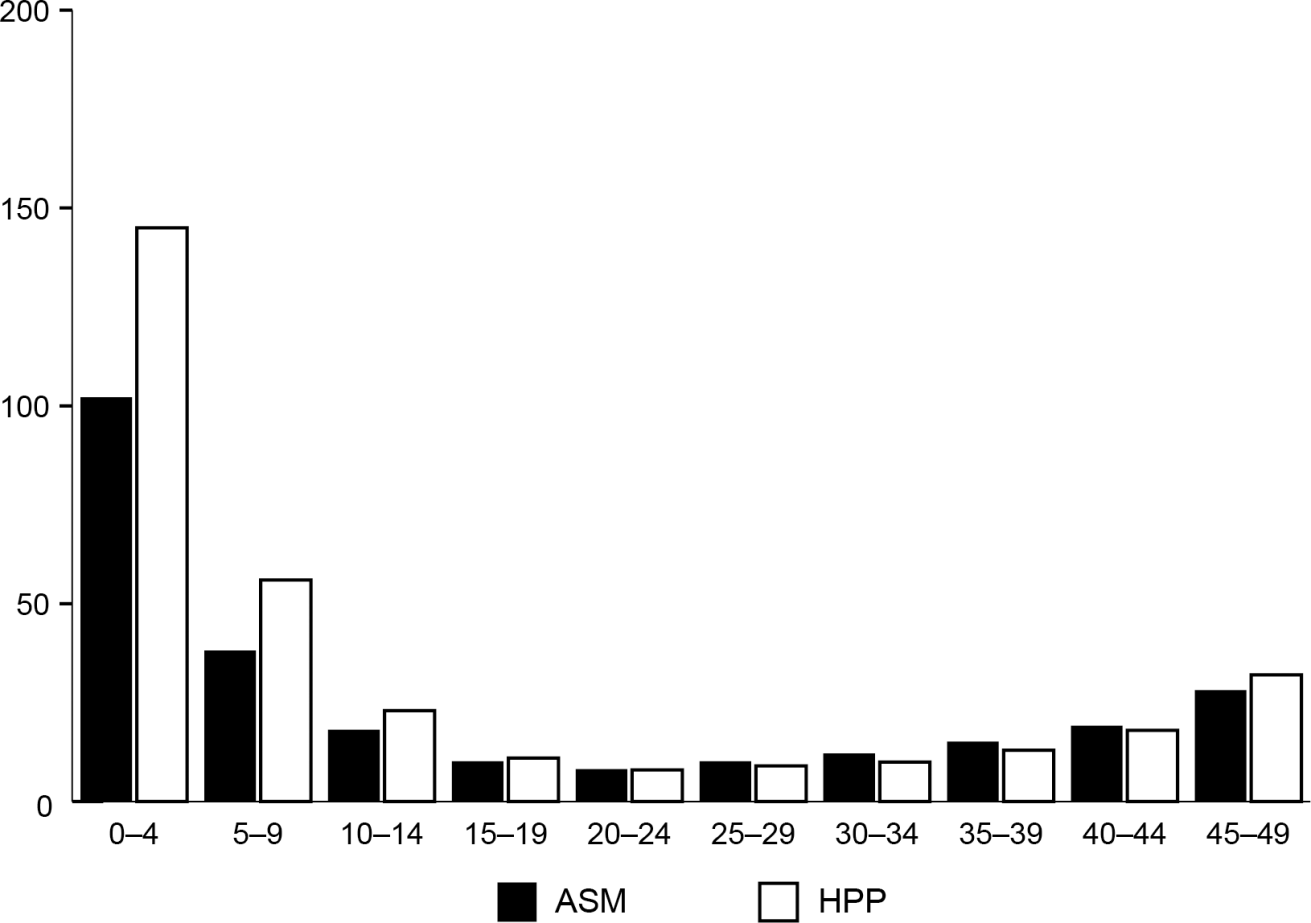
Comparison of results from the DMPPT 2.0 and ASM models. The figure shows the number of VMMCs per HIV infection averted in each age group in Zambia, 2010–2025. Hypothetical scenarios were run, in which circumcision prevalence was scaled up to an 80% coverage level one five-year age group at a time. HIV infections averted are projected across the entire population.

This model was intended to be relatively easy to use so that it could be readily applied by national program personnel to investigate the variable epidemiological impact and cost of VMMC scale-up by age at circumcision at national or subnational levels. The model also produces results that are comparable to those obtained by the more complex ASM model [26]. When data from Zambia were analyzed with both the DMPPT 2.0 and ASM models and the same HIV incidence projections were used for both models, the numbers and pattern of VMMCs required to avert one HIV infection by client age-group were found to be similar (Fig 2). In the absence of this standardization, because of differences in how the models derive the age distribution of HIV incidence, the ASM model tended to prioritize slightly lower age groups than the DMPPT 2.0 did.

The DMPPT 2.0 model’s simplicity does impose the following limitations:

- Although future HIV incidence rates change with time, the pattern of HIV incidence by age is assumed to remain constant in the future in the absence of scaled-up VMMC programs. In reality, the pattern may change due to expanded treatment eligibility, improved population-level viral suppression, viral resistance, scale-up of other prevention interventions, or secular trends. This may result in overestimating or underestimating impact.
- The mortality rate is based on total mortality (HIV-related mortality and non-HIV-related mortality) from the official national projection. In reality, the mortality rate may change as the scaling up of VMMC affects HIV prevalence. This is unlikely to have significant effects over the short term (five to seven years) but the effects will grow with longer periods.
- The model captures the primary effects (HIV infections averted among men who are circumcised), and estimates the secondary effects (HIV infections averted among female partners of circumcised men) but it does not capture tertiary effects (men who are not circumcised but receive some protection because of lower HIV prevalence among their female partners). The model also does not capture prevention benefits indirectly associated with accessing VMMC services, including the benefits of HIV counseling and testing, prevention, and treatment of sexually transmitted infections and the benefits of early referral to antiretroviral treatment (ART) services for HIV-positive VMMC clients.
- This model does not include the impact on HIV incidence of other interventions that might be scaled up at the same time, such as ART or condom promotion. The impact of those interventions on HIV incidence has to be modeled separately using the Spectrum/Goals [29] model and then transferred to the DMPPT 2.0. The impact and cost of scaling up VMMC in the context of ART scale-up is explored in another manuscript in this collection [30].

### Data used

The data used for the modeling of age targeting of VMMCs in Malawi, South Africa, Swaziland, Tanzania, and Uganda are described in detail in accompanying manuscripts in this collection [21–25]. All model inputs can be found in the supplemental materials (S1 Appendix). Briefly, population by age and year, mortality by age and year, annual number of male births, and HIV prevalence by age and year were exported from Spectrum/Goals or Spectrum/AIM files for all countries (see individual country application manuscripts for details for each country). The HIV incidence was also obtained from Spectrum/Goals or Spectrum/AIM files for Malawi, South Africa, Tanzania, and Uganda. For Swaziland, the age-specific HIV incidence was derived from the Swaziland Incidence Measurement Survey (SHIMS) [31]. The MC prevalence by age group in the model base years was derived from household surveys [32–36]. The unit costs of VMMC used for each country were based on expenditure analyses conducted by the PEPFAR country teams and were $100, $125, $109, $83, and $80 U.S. dollars (USD) for Malawi, South Africa, Swaziland, Tanzania, and Uganda respectively [21–25]. A discount rate of 3% was used both for costs and HIV infections averted. All costs hereafter are presented in 2014 U.S. dollars.

### Uncertainty analysis

Uncertainty around the age distribution of HIV incidence was calculated as follows: for each age group, the HIV incidence for every year of the model was multiplied by a random number between 0.85 and 1.15, based on the estimated 15% variance in HIV incidence by age from the Analysing Longitudinal Population-based HIV/AIDS data on Africa (ALPHA) Network [37] incidence rate ratios by age used in Spectrum/AIM. Because the HIV incidence for the Swaziland model was derived from the SHIMS study [31] rather than from Spectrum, for the Swaziland uncertainty analysis the uncertainty around the age distribution was derived from the confidence intervals around each age-specific incidence estimate from the SHIMS study. The Swaziland HIV incidence for each age group was varied randomly between the lower 95% confidence bound for HIV incidence in that age group and the upper 95% confidence bound for HIV incidence in that age group. Uncertainty bounds around age-specific model outputs that depend on HIV incidence are produced by generating each model output 100 times using the random variation described above for the age distribution of HIV incidence, and then taking the mean plus or minus 1.96 times the standard deviation of the 100 model outputs.

### Framework for analyzing VMMC age-targeting strategies

The DMPPT 2.0 and ASM developers and experts from USAID, the U.S. Centers for Disease Control and Prevention (CDC), the U.S. Department of Defense (DOD), the Office of the U.S. Global AIDS Coordinator (OGAC), BMGF, the World Health Organization (WHO), the United Nations Children’s Fund (UNICEF), the Joint United Nations Programme on HIV/AIDS (UNAIDS), and the World Bank developed an analytical framework in October 2013 to assess the impact and cost of VMMC age-targeting strategies in the countries studied. This framework comprises the following four metrics: 1) efficiency, defined as the number of VMMCs per HIV infection averted over 15 years (a 15-year period was chosen as it was considered to be a short enough time to be relevant to policymakers but long enough to accrue some of the prevention effects of VMMC); 2) immediacy of impact, which refers to the reduction in HIV incidence over five years, as compared with a scenario in which VMMC remains at baseline levels; 3) magnitude of impact, which is the reduction in HIV incidence over 15 years when compared to baseline VMMC levels; and, 4) cost-effectiveness, which is defined as the cost per HIV infection averted over 15 years.

For each of the analyses in the framework, circumcision prevalence in the indicated age group was scaled up to 80% coverage levels between 2014 and 2018, and then maintained at that coverage level after 2018. The number of HIV infections averted was projected over the entire population (not only within the indicated age group).

## Results

### Efficiency of VMMC age-targeting strategies

**Fig 3:**
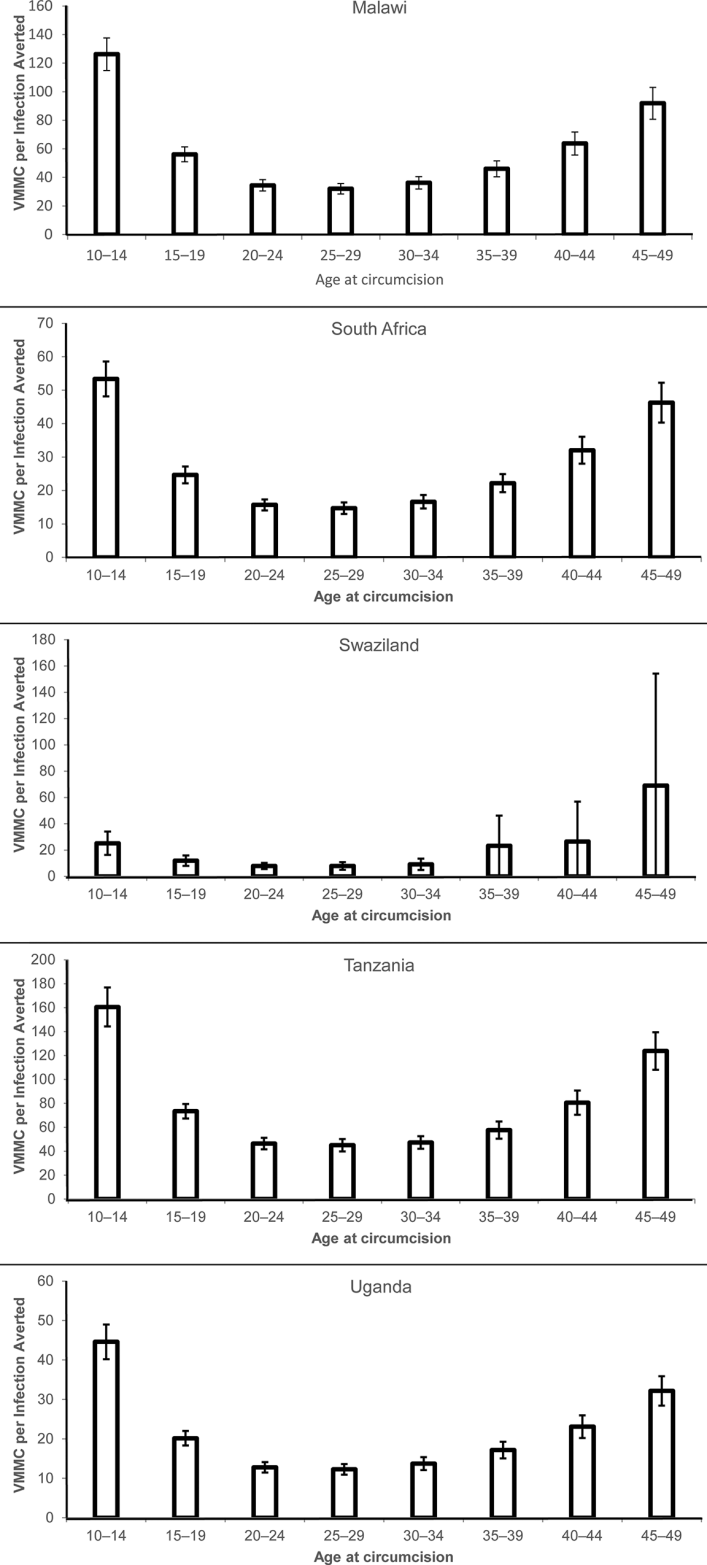
VMMCs per HIV infection averted by country, 2014–2028. Each bar represents a scenario in which circumcisions are performed only in the specific age group depicted, and circumcision prevalence is scaled up to 80% within that age group. HIV infections averted are projected across the entire population.

Hypothetical scenarios were run, in which circumcision prevalence was scaled up to an 80% coverage level one five-year age group at a time. Fig 3 shows the number of VMMCs per HIV infection averted by five-year age groups in Malawi, South Africa, Swaziland, Tanzania, and Uganda. The lowest number of VMMCs per HIV infection averted was produced by circumcising 20- to 24-, 25- to 29-, and 30- to 34-year-olds in Malawi, South Africa, Tanzania, and Uganda and 15- to 19-, 20- to 24-, 25- to 29-, and 30- to 34-year-olds in Swaziland, although the uncertainty bounds for the estimates overlap due to the wide confidence intervals around the HIV incidence estimates from the SHIMS study.

### Immediacy and magnitude of impact of VMMC age-targeting strategies

**Fig 4:**
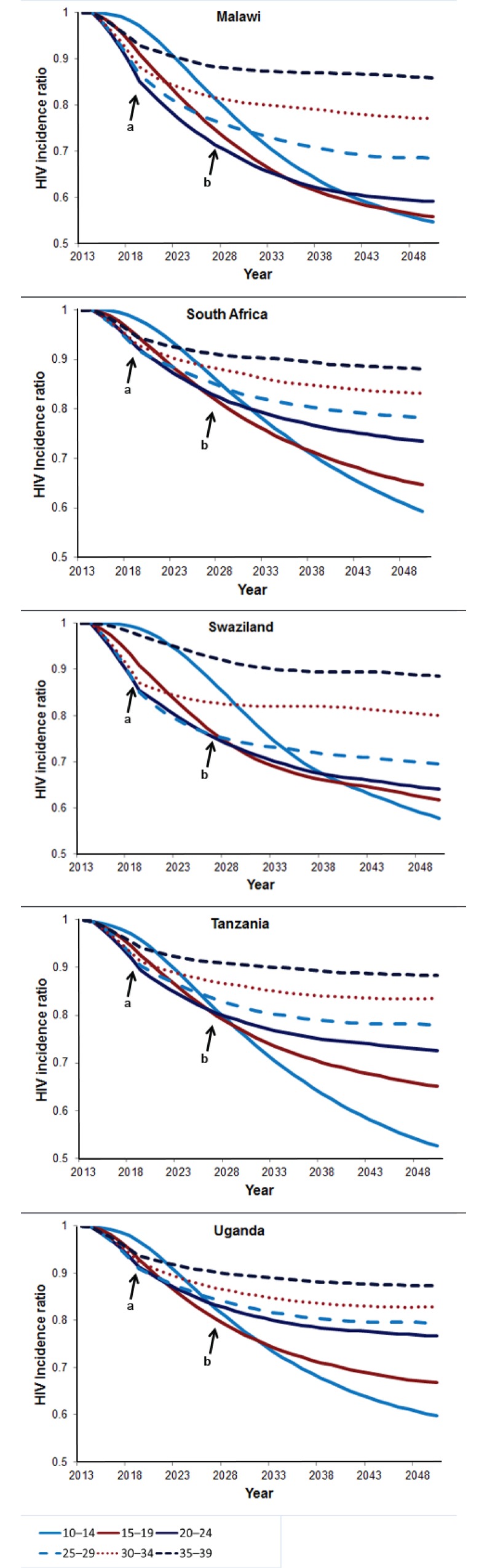
HIV incidence ratios by country, 2014–2048. (a) immediacy of impact (five years). (b) magnitude of impact (15 years). HIV incidence in VMMC scale-up scenarios in which circumcisions are only provided to the five-year age group indicated, divided by HIV incidence in a scenario in which the baseline male circumcision prevalence by age is maintained. 1 = the HIV incidence in the baseline scenario in each year.

Fig 4 (reproduced from country manuscripts [21–25]) depicts the impact of VMMC on HIV incidence in the short (immediacy of impact) and medium terms (magnitude of impact). Each line corresponds to a hypothetical scenario in which circumcision prevalence was scaled up to an 80% coverage level within one five-year age group alone. When considering only the period 2014–2018, the most rapid reductions in HIV incidence can be seen in the strategies focused on 20- to 24- and 25- to 29-year-olds in Swaziland and on 20- to 24-, 25- to 29-, and 30- to 34-year-olds in Malawi, South Africa, Tanzania, and Uganda. In contrast, when considering a 15-year timeframe (2014–2028), the reduction in HIV incidence flattens out at a level related to the fraction of lifetime protection conferred by circumcising at a given age. The greatest reductions in HIV incidence after 15 years can be seen in the strategies focused on 10- to 14- and 15- to 19-year-olds in Uganda; on 15- to 19- and 20- to 24-year-olds in Malawi and South Africa; on 10- to 14-, 15- to 19-, and 20- to 24-year-olds in Tanzania; and on 15- to 19-, 20- to 24-, and 25- to 29-year-olds in Swaziland. When considering the even longer perspective of 2014–2048, circumcising 10- to 14-year-old males confers the greatest long-term benefits, as they are protected from HIV for their entire lifetime of sexual exposure to HIV, and these benefits accrue over time to the males themselves and to their sexual partners. Conversely, males circumcised in the higher age groups have less time to benefit from the protection of circumcision.

### Cost-effectiveness of VMMC age-targeting strategies

To assess the cost-effectiveness of different age targeting strategies, scenarios were also run scaling up VMMC to target coverage levels for groups with age ranges wider than five years (10–49, 15–49, 15–24, 15–29, 15–34, 10–24, 10–29, and 10–34), to better explore potential priority age groups for actual scale-up strategies. The HIV infections averted, the number of VMMCs needed during the five-year scale-up phase, the cost per HIV infection averted, and the total cost of VMMC scale-up and maintenance over the 15-year period (assuming that cost does not vary by age of client) are shown in Table 1. Uncertainty analyses for cost-effectiveness and HIV infections averted, based on the uncertainty in age-specific HIV incidence, are presented elsewhere [21–25]. In addition, sensitivity analyses assessing the effects of varying the VMMC unit cost by client age are presented in [23] and [38].

**Table 1:** Impact, cost-effectiveness, and total cost of VMMC scale-up by target age group, 2014–2028. Reference scenario is one in which male circumcision is not scaled up above baseline levels prior to the start of the VMMC program.

		Malawi	South Africa	Swaziland	Tanzania	Uganda
HIV infections averted, thousands (2014–2028)	10–49	149	375	31.5	53.4	486
	15–49	148	372	32.2	50.5	475
	15–24	82	182	18.9	28.3	241
	15–29	109	246	25.7	36.2	324
	15–34	128	303	29.7	43.2	388
	10–24	83	190	19.6	31.3	256
	10–29	110	250	26.3	38.7	337
	10–34	129	307	29.9	45.9	403
# scale-up VMMCs, millions (2014–2018)	10–49	4.8	7.2	0.27	2.6	6.3
	15–49	3.7	5.6	0.21	1.8	4.6
	15–24	1.9	2.5	0.11	0.9	2.1
	15–29	2.4	3.2	0.15	1.1	2.7
	15–34	2.9	4.0	0.18	1.4	3.3
	10–24	3.0	4.0	0.16	1.7	3.8
	10–29	3.6	4.8	0.20	1.9	4.4
	10–34	4.0	5.6	0.23	2.2	5.0
Discounted cost per HIV infection averted, thousands in USD (2014–2028)	10–49	$4.6	$2.7	$1.2	$5.8	$1.5
	15–49	$3.5	$2.2	$0.9	$4.1	$1.1
	15–24	$4.3	$2.5	$1.0	$4.9	$1.4
	15–29	$3.7	$2.2	$0.9	$4.3	$1.2
	15–34	$3.5	$2.1	$0.9	$4.0	$1.1
	10–24	$6.1	$3.6	$1.4	$7.8	$2.1
	10–29	$5.1	$3.0	$1.2	$6.8	$1.7
	10–34	$4.7	$2.7	$1.1	$6.1	$1.6
Total cost, millions in USD (2014–2028)	10–49	$676	$1,021	$37	$309	$723
	15–49	$522	$806	$29	$204	$523
	15–24	$352	$458	$18	$139	$330
	15–29	$406	$541	$22	$156	$379
	15–34	$449	$627	$25	$174	$428
	10–24	$506	$673	$26	$243	$531
	10–29	$559	$755	$30	$261	$581
	10–34	$602	$841	$33	$278	$629

The greatest number of HIV infections averted is achieved by circumcising the largest age group in the model—10- to 49-year-olds—in all countries except Swaziland, where the number of HIV infections among this age group is slightly smaller than that for those ages 15–49, because of the effects of discounting. (The reader is reminded that the Swaziland age-specific HIV incidence came from the SHIMS study, while for the other countries it was exported from the Spectrum model. Therefore, the age distribution of HIV incidence in the Swaziland model was markedly different than that of the other countries analyzed. When the discount rate was set to zero in the Swaziland model, the number of HIV infections averted was greater when circumcising the 10- to 49-year-olds than the 15- to 49-year-olds. In either case, the differences in HIV infections averted between the two age groups are smaller than the uncertainty around the estimates, as shown in Fig 2 in [25].) Focusing the scale-up of VMMC coverage on some of the younger age groups would result in nearly as many HIV infections averted as scaling up coverage among all 15- to 49-year-olds. For example, scaling up VMMC among 10- to 34-year-old males would result in 83% to 93% of the HIV infections that would be averted by scaling up coverage to 15- to 49-year-olds, while scale-up of coverage to 10- to 29-year-old males would result in 67% to 82% of the HIV infections averted resulting from achieving the coverage targets among 15- to 49-year-olds. In South Africa and Tanzania, the lowest cost per HIV infection averted would be achieved by circumcising males ages 15–34; in Malawi and Uganda, the lowest cost per HIV infection averted would be attained by focusing either on males ages 15–34 or ages 15–49; and in Swaziland, focusing on the age groups 15–29, 15–34, or 15–49 would be most cost-effective.

## Discussion

Results of DMPPT 2.0 modeling of age-targeting strategies in Malawi, South Africa, Swaziland, Tanzania, and Uganda showed that the efficiency, immediacy of impact, magnitude of impact, and cost-effectiveness of VMMC scale-up is not uniform; there is important variation by the age group of the males circumcised. Though there was overlap, the age ranges identified as priorities differed somewhat across the four metrics and the countries. This suggests that VMMC scale-up in each of the five countries studied should be modified from the initial target of 15- to 49-year-olds set out in the Joint Strategic Action Framework to Accelerate the Scale-Up of Voluntary Medical Male Circumcision for HIV Prevention in Eastern and Southern Africa [16]. The lowest number of VMMCs per HIV infection averted would be produced by circumcising males ages 20–34 in Malawi, South Africa, Tanzania, and Uganda and males ages 15–34 in Swaziland. The most immediate impact on HIV incidence would be generated by circumcising males ages 20–34 in Malawi, South Africa, Tanzania, and Uganda and males ages 20–29 in Swaziland. The greatest reductions in HIV incidence over a 15-year period are achieved by strategies focused on males 10–19 in Uganda, 15–24 in Malawi and South Africa, 10–24 in Tanzania, and 15–29 in Swaziland. Focusing on the age group 15–34 is one of the most cost-effective options in all countries.

The strength of the DMPPT 2.0 model is that it allows policymakers to examine the epidemiological impact, relative cost, and cost-effectiveness of circumcising different age groups of clients in a way that is relatively easy to understand and use. Countries can readily use it as a planning tool to examine the consequences of different scale-up scenarios by age group.

Like any model, this one is limited by the quality of the data and the validity of the assumptions underlying it, such as the baseline male circumcision prevalence by age, the effectiveness of male circumcision in preventing HIV acquisition, the projection of HIV incidence into the future, the age and geographic distribution of HIV incidence estimates, and the unit costs of VMMC and ART. In conducting country applications of the model, we met with policymakers, donors, and other stakeholders in each country to review and adjust the assumptions, to ensure that the model application reflected the best current data and understanding, and also to ensure that the country stakeholders fully understood the model’s limitations.

An update to the DMPPT 2.0 model, called DMPPT 2.1, has recently been completed; it includes additional features that will help countries assess the impact of their programs to date, to easily examine more flexible scale-up scenarios, to use the model as a monitoring tool, and to adjust their targets as conditions change [18].

While the results of the model are internally consistent and appropriate for setting priorities within the VMMC program, it is not advisable to use the “total cost” projections from the model to estimate actual program costs, for a number of reasons. First, the unit costs used in the model typically combine costs incurred in different time frames and from different cost centers. For example, equipment and construction costs are usually amortized when calculating a unit cost, but a funder may pay for these costs all at once. Unit costs may include government and donor costs, or costs that come from different portions of a government’s budget. Second, an actual VMMC program will include different implementation models, such as fixed and mobile sites, and costs likely differ by implementation model and whether the activities are conducted in urban areas or difficult-to-reach areas of low population density. Last, the model is not set up to reflect how costs may change over time as a program matures. Therefore, VMMC program budgeting should be conducted through a separate process that accounts for the actual activities and commodities that will need to be funded through a specific funding stream.

Because of its structural simplicity, this model cannot be used to answer questions about the impact of changing patterns of sexual behavior, differential representation of HIV-positive people among the population of VMMC clients, changes over time in costs of VMMC and ART, the impact of scaling up VMMC on human resources for health, the impact of VMMC in the context of changing coverage of other prevention interventions, and other questions that may be of interest to policymakers. Some of these questions, such as those concerning risk behavior and differential representation of HIV-positive people in the client population, can be addressed using the ASM [26].

Other questions, such as the impact of VMMC in the context of changing coverage of ART and other prevention interventions, or the impact of changing costs for VMMC and ART over time, can be examined using the Spectrum/Goals model [29], another sophisticated compartmental model that incorporates information about epidemiology, behavior, HIV mortality and disease progression, and intervention effectiveness. The Goals model allows the user to examine scenarios in which coverage and cost of a variety of HIV prevention and treatment interventions can be varied over time, and the model returns the impact of HIV-related deaths, new HIV infections, costs, and other metrics for each scenario. Another manuscript in this collection [30] explores the impact of scaling up VMMC in the context of the new UNAIDS 90-90-90 targets for treatment [39]. The Goals, ASM, and DMPPT 2.0 models have different purposes and complement one another.

### Policy implications of circumcising adolescents ages 10–14

The DMPPT 2.0 modeling done to date shows that focusing on the age group 15–34 is one of the most cost-effective options in all countries studied. *Over a 15-year time frame*, inclusion of males ages 10–14 only leads to a small increase in HIV infections averted, and results in a higher cost per HIV infection averted. This is because the majority of males ages 10–14 are not highly sexually active, so they must wait to accrue the benefit of circumcision later, when they have a higher risk of exposure to HIV. The model shows that over the long term (30+ years), circumcising males ages 10–14 delivers the greatest reduction in HIV incidence from VMMC compared with other age groups. While an analysis focused on short-term impact might lead one to conclude that circumcising males ages 10–14 should not be a program priority, turning these individuals away would mean refusing services to approximately 35% of clients currently accessing VMMC services [18] and is viewed as problematic by implementers, given the limited demand for VMMC to date among older males. Such a policy would likely have unintended consequences, such as loss to follow-up, or worse, creating a negative perception of VMMC within the community. It is also worth noting that circumcising males ages 10–14 is likely to be more cost-effective than circumcising older males, who appear to require more complex demand-creation efforts [40]. Furthermore, male circumcision, unlike any other HIV prevention intervention, is an intervention that only needs to be provided once and leads to a lifetime benefit. Thus, analyses that consider only short-term benefits miss the point of this highly cost-effective HIV prevention method whose effects last a lifetime.

### Policy implications of circumcising older men

At the time that development of this model was initiated, VMMC implementers had realized that it was difficult to recruit men over the age of 25, and particularly difficult to recruit those above age 35. The conventional wisdom at the time stated that because intergenerational sex was a driver of the epidemic in several African countries, circumcising men ages 30–40 would be key to interrupting transmission. This analysis shows that circumcising older men does not result in relatively large reductions in HIV incidence in women, as most men will have acquired the virus at younger ages. So the best way to reduce HIV prevalence among older men, and thus protect their younger female partners, is to circumcise the men at younger ages, before they reach the age of peak HIV incidence and before they become sexually active. As with males ages 10–14, policymakers in the countries where we have applied the model do not wish to turn older men away from services if they are medically eligible and will continue to provide VMMC services to those who want them.

The DMPPT 2.0 model was created to examine the effects of client age and geographic region on the cost-effectiveness and epidemiological impact of VMMC programs. Model results, combined with implementation realities, have implications for how VMMC programs approach adolescents ages 10–14 and men over age 30. More information is needed regarding the cost and effectiveness of demand creation and services focused on different age groups of clients.

## Supporting Information

S1 Appendix DMPPT 2.0 Model Inputs. (XLSX)

## Abbreviations

UNAIDS: Joint United Nations Programme on HIV/AIDS
PEPFAR: President’s Emergency Plan for AIDS Relief
VMMC: voluntary medical male circumcision
DMPPT: Decision Makers’ Program Planning Toolkit
USAID: United States Agency for International Development
BMGF: Bill & Melinda Gates Foundation
ASM: Age-Structured Model
AIM: AIDS Impact Model
ART: antiretroviral therapy
SHIMS: Swaziland HIV Incidence Measurement Survey
MOH: Ministry of Health
USD: U.S. dollars
ALPHA: Analysing Longitudinal Population-based HIV/AIDS Data on Africa
CDC: U.S. Centers for Disease Control and Prevention
DoD: U.S. Department of Defense
OGAC: Office of the U.S. Global AIDS Coordinator
WHO: World Health Organization
UNICEF: United Nations Children’s Fund
PPD ARO: Partners in Population and Development, Africa Regional Office
PRB: Population Reference Bureau
WRA: White Ribbon Alliance
