# Correction: Age Targeting of Voluntary Medical Male Circumcision Programs Using the Decision Makers' Program Planning Toolkit (DMPPT) 2.0

**DOI:** 10.1371/journal.pone.0174466

**Published:** 2017-03-16

**Authors:** Katharine Kripke, Marjorie Opuni, Melissa Schnure, Sema Sgaier, Delivette Castor, Jason Reed, Emmanuel Njeuhmeli, John Stover

The image for Fig 1 is incorrect. Please see the complete, correct Fig 1 here.

**Fig 1:**
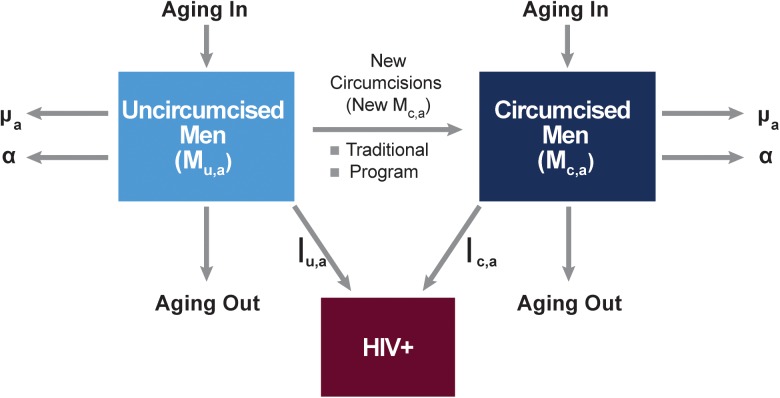
Structure of the DMPPT 2.0 model. μ, non-AIDS mortality; α, AIDS-related mortality; *I*_*u*,*a*,_, HIV incidence of uncircumcised men; *I*_*c*,*a*_, HIV incidence of circumcised men.
